# A case of Tinea Faciei caused by *Trichophyton benhamiae*: first report in China

**DOI:** 10.1186/s12879-020-4897-z

**Published:** 2020-02-22

**Authors:** Jingwen Tan, Xiaoping Liu, Zhiqin Gao, Hong Yang, Lianjuan Yang, Hai Wen

**Affiliations:** 1 Department of Medical Mycology, Shanghai Dermatology Hospital, Shanghai, 200443 China; 2 Department of Dermatology, Shanghai Changzheng Hospital, Second Affiliated Hospital of Naval Military Medical University, Shanghai, 200003 China

**Keywords:** *Trichophyton benhamiae*, Tinea faciei, Terbinafine, Fox, Case report

## Abstract

**Background:**

*Trichophyton benhamiae* is a zoophilic dermatophyte that can cause tinea in humans and animals. Lesions caused by *T. benhamiae* tend to be highly inflammatory, and patients are often infected by animals or other patients infected with *T. benhamiae*. In this paper, we report the first case of tinea faciei caused by *T. benhamiae* in a Chinese girl who might be transmitted from a fox.

**Case presentation:**

A 4-year-old girl from HaiNing city developed an itchy, erythematous, and annular plaque on her right face for the past 2 months. Before the lesion appeared, she was in close contact with the fur of a fox for almost 1 week. Septate hyaline hyphae were detected by direct mycological examination of the scales. Cultures grew on Sabouraud’s dextrose agar (SDA) at 26 °C for 2 weeks revealed the presence of *T. mentagrophytes*. A molecular sequencing test confirmed that the isolate was consistent with reference strains to *T. benhamiae*. Then, the diagnosis of tinea faciei due to *T. benhamiae* was made. Treatment with terbinafine (oral 125 mg/d) and sertaconazole nitrate cream (topical, twice daily) for 4 weeks was initiated and achieved significant improvement of the skin lesions.

**Conclusions:**

This rare dermatophytosis case highlights the importance of ITS sequencing in helping to recognize rare pathogenic fungi that can be easily misdiagnosed with a conventional morphological diagnosis.

## Background

*Trichophyton benhamiae* is a zoophilic dermatophyte that can cause highly inflammatory tinea in humans and animals [1]. Guinea pigs are the primary carrier, and other small animals are occasionally a source of infection [2]. Due to the increased variety of pets, *T. benhamiae* infection is rising. Cases caused by *T. benhamiae* infection had been reported in several countries such as Japan, Germany, or Switzerland [1]. In Germany, *T. benhamiae* is the most prevalent pathogen causing zoophilic dermatophytosis, especially in children [3]. Here we report a case of tinea faciei caused by *T. benhamiae* that might have been transmitted by a fox. To the best of our knowledge, this is the first report of dermatophytosis caused by *T. benhamiae* in China.

## Case presentation

A 4-year-old girl from HaiNing city developed an itchy, erythematous, and annular plaque on her right face for the past 2 months. The lesion was initially tiny erythema with scale. Topical clobetasol propionate ketoconazole cream was administered for 15 days without any response. The treatment was switched to pimecrolimus and hydrocortisone butyrate cream, but the lesion became lightly tender, itchy, and transforming into a “ring” erythematous plaque. The patient attended to our clinic in December 2018. Her mother denied any previous history of trauma. A remarkable antecedent was the fact that her family feeds foxes as a source of income. Before the lesion appeared, she was in close contact with the fur of a fox for almost 1 week. The rest of her medical and family history was unremarkable.

**Fig. 1:**
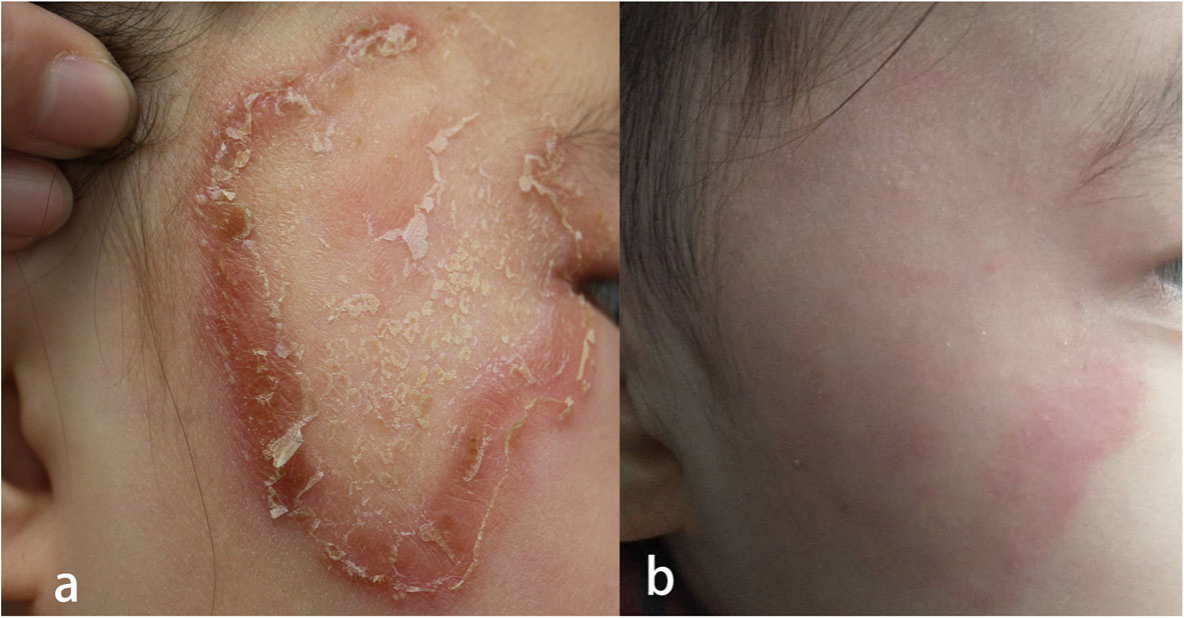
Clinical appearance. **a** Annular plaque with erythema covered with scales and crusts (3 cm × 5 cm) on the right face. **b** Complete resolution of the plaque with residual erythema after 4 weeks of treatment with terbinafine (oral, 125 mg/d)

**Fig. 2:**
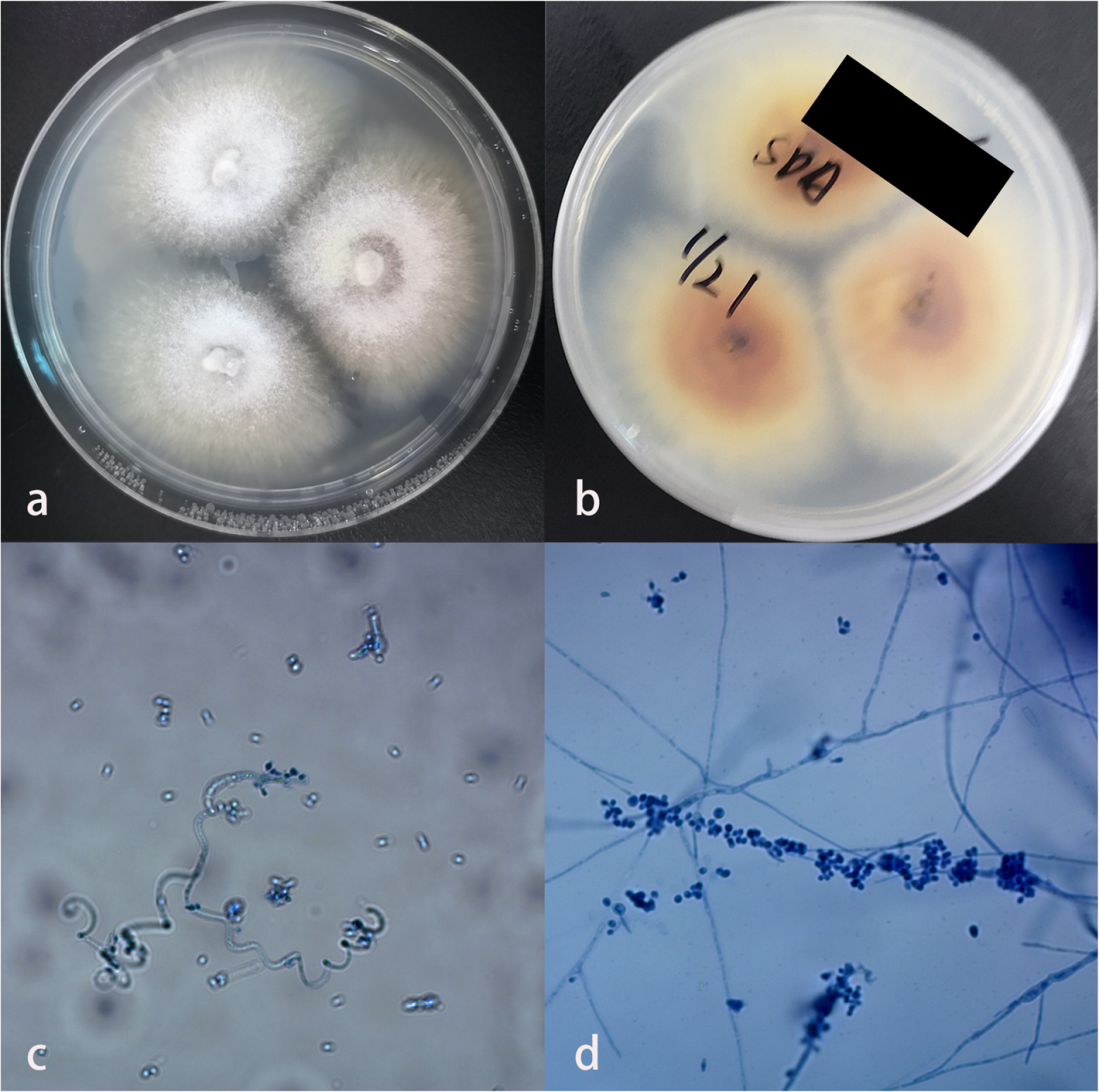
Mycological findings. **a** and **b** Culture on SDA at 26 °C after 2 weeks yielded white colonies, peripherally radiating, centrally raised, and powdery margins. The reverse side showed yellow to brown colonies. **c** Lactophenol cotton blue stain revealed filamentous and spiral hyphae (original magnification ✕ 200). **d** Lactophenol cotton blue stain revealed a grape-like arrangement of microconidia laterally and terminally inserting at the hyphae (original magnification ✕ 200)

The physical examination showed a 3 cm × 5 cm erythematous annular plaque on her right face covered with scales and crusts (Fig. 1a). Regional lymph nodes were not palpable. Direct mycological examination by lesion scraping with 10% KOH showed the presence of septate hyaline hyphae. A sample was cultured on sabouraud’s dextrose agar (SDA) at 26 °C for 2 weeks that yielded white colonies peripherally radiating, centrally raised, and powdery margins (Fig. 2a). The reverse side showed colonies with a color yellow to brown (Fig. 2b). Slides from the culture showed filamentous and spiral hyphae with a grape-like arrangement of the microconidia laterally and terminally inserting at the hyphae (Fig. 2c, d). Based on the morphological characteristics, the isolate was identified as *T. mentagrophytes*. Then molecular sequencing of the internal transcribed spacer (ITS) region gene was performed. Briefly, genomic DNA extracted from the culture employing Ezup Column Fungi Genomic DNA Purification Kit (Sangong Biotech, Shanghai) according to the manufacturer’s instructions. Then PCR reaction was carried out to amplify the ITS Region with the following primers: ITS1 (5′TCCGTAGGTGAACCTGCGG) and ITS4 (5′-TCCTCCGCTTATTGATATGC). Amplification was performed on a Veriti (Applied Biosystem) with following conditions: denaturation at 94 °C for 5 min, followed by 30 cycles of 94 °C for 30s, 54 °C for 30s, 72 °C for 60s, and finally an extension 72 °C for 8 min. After verified by electrophoresis on 1.0% agarose gels, the PCR-amplified product was sent to Sangon Biotech (Shanghai) for sequencing. Sequence of this isolate determined in this study was aligned with reference sequences in genbank (https://blast.ncbi.nlm.nih.gov/Blast.cgi). A comparison of the ITS (648 bp) (genbank accession number MN536486) sequence with the genbank database revealed 100% similarity with *T. benhamiae* reference strain ATCC42873 (genbank accession number KX092365.1). The fungal culture was finally identified as *T. benhamiae*. The girl was diagnosed with tinea faciei caused by *T. benhamiae*.

In vitro susceptibility was tested following the Clinical and Laboratory Standards Institute (CLSI) M38-A2 protocol [4]. The minimum inhibitory concentration (MIC) was determined by 100% inhibition compared with the growth control. The results revealed that all the tested drugs were susceptible to the isolate. The MIC values was 4 μg/ml for fluconazole (FLZ), 1 μg/ml for itraconazole (ITC), 0.6 μg/ml for voriconazole (VRC) and posaconazole (POS), 1 μg /ml for caspofungin (CAS), and 0.015 μg/ml for terbinafine (TRB). Oral administration of terbinafine (125 mg/day) and topical sertaconazole nitrate cream (twice daily) was initiated, significant improvement of the lesions was achieved after 4 weeks of treatment. No adverse event was reported.

## Discussion and conclusions

*T. benhamiae* (previously know as *Arthroderma benhamiae*) was first described as teleomorph of the *T. mentagrophytes* complex in 1967 [5]. In the latest dermatophyte taxonomy based on the sequencing of the ITS ribosomal DNA region, *Trichophyton* sp. of *A. benhamiae* does not belong to the *T. mentagrophytes* complex anymore; it became *T. benhamiae* [6].

The first human tinea corporis caused by this fungus was reported in 1975; it was a case of a lab worker that got a hand infection after contact with an infected hedgehog three times in 3 weeks [7]. Since then, *T. benhamiae* has been diagnosed more frequently around the world. Until now, there are 30 human case reports of *T. benhamiae* infection confirmed by molecular methods (Table 1). Interestingly, 30% of them (10/30) were family members or lived together. The patients’age ranged from 19 months to 53 years old and were spread in three continents (Asia, Europe, and South America). 18/29 of them were under 18 years old, and Tinea corporis was the most common type (15/30) followed by Tinea faciei (13/30). Guinea pigs are the most common source (16/28) of this pathogen, followed by other small animals such as rabbits, cats, and dogs. In this case, the infection source was from a fox. Even though foxes can be a host of *T. benhamiae*, there was no previous report of human infection by fox until now [23]*.*

**Table 1:** Cases of *T. benhamiae* infection confirmed with molecular sequencing

No	Country	Year	Sex	Age	Diagnosis	Animal	Treatment	Outcome	Reference	Note
1	England	1975	/	/	Tinea corporis	Hedgehog	Topical: Cetrimide; Iodine ointment; Nystatin	Cure	[7]	
2	Japan	1996	F	7	Tinea corporis	Rabbit	Topical: Lanoconazole	Cure	[8]	Daughter and Mother
3	Japan	1996	F	30	Tinea corporis	Rabbit	Topical: Butenafine	Cure		
4	Japan	2000	F	4	Kerion celsi	Guinea pig	Oral: ITC; Topical: Lanoconazole	Cure	[9]	
5	Japan	2002	M	29	Tinea corporis	Rabbit	Topical: Ketoconazole	Cure	[10]	Couple
6	Japan	2002	F	31	Tinea corporis	Rabbit	Topical: Ketoconazole	Cure		
7	Japan	2002	F	53	Tinea faciei	Laboratory	Topical: Butenafine	Cure	[11]	
8	Switzerland	2002	F	13	Tinea faciei	Hamster	/	/	[12]	
9	Switzerland	2002	F	13	Tinea corporis	Dog, Cat, Rabbit, Chicken	/	/		
10	Switzerland	2002	F	17	Tinea corporis	/	/	/		
11	Switzerland	2002	F	14	Tinea faciei	Guinea pig	/	/		
12	Switzerland	2002	F	11	Tinea faciei	Guinea pig	/	/		
13	Switzerland	2002	F	12	Tinea faciei	Guinea pig, Horse	/	/		
14	Switzerland	2002	F	33	Tinea corporis	Guinea pig	/	/		
15	Switzerland	2002	F	12	Tinea corporis and faciei	Guinea pig	/	/		
16	Switzerland	2002	F	8	Tinea corporis	Guinea pig, Rabbit	/	/		
17	Japan	2006	F	25	Tinea corporis	Rabbit	Oral:TRB; Topical: Ketoconazole	Cure	[13]	
18	Japan	2010	F	27	Tinea corporis	Guinea pig	Oral: TRB; Topical: Ketoconazole	Cure	[14]	Sisters
19	Japan	2011	F	25	Tinea faciei	Guinea pig	Topical: Liranaftate	Cure		
20	Germany	2013	M	24	Tinea faciei	Guinea pig	Antimycotic therapy	Cure	[15]	
21	Germany	2013	M	9	Kerion celsi	Guinea pig	Oral:TRB	Cure	[16]	
22	Japan	2013	F	36	Tinea faciei	**/**	Oral:TRB; Topical: Clotrimazole	Cure	[17]	
23	UK	2016	F	2	Tinea corporis	Guinea pig	Topical: TRB	Cure	[18]	
24	Brazil	2016	F	19 month	Tinea corporis	Cat	Systemically: Griseofulvin; Topical: Butenafine, TRB	Cure	[19]	
25	Korea	2018	F	46	Tinea faciei	Rabbit	Oral: TRB	Cure	[20]	Mother and Daughter
26	Korea	2018	F	8	Tinea faciei	Rabbit	Oral: TRB	Cure		
27	Span	2019	F	3	Kerion celsi	Guinea pig	Oral: ITC; Topical: Miconazole	Cure	[21]	
28	Span	2019	F	8	Tinea corporis	Guinea pig	Oral and topical TRB	Cure		
29	Italy	2019	F	9	Tinea faciei	Guinea pig	Oral: TRB	Cure	[22]	Sister and Brother
30	Italy	2019	M	3	Tinea faciei	Guinea pig	Oral: TRB	Cure		

/ Undetected, *F* Female, *M* Male, *ITC* Itraconazole, *TRB* Terbinafine

This pathogen had white and yellow phenotypes, which can difficult its identification [24]. The micromorphology of yellow colonies is downy with a pleated mycelium and a slow growth rate. They can have a rough-walled and spindle-like macroconidia. The most common differential diagnosis of the yellow phenotype is *Microsporum canis.* The micromorphology of white colonies is powdery to floccose, and with a rapid growth rate. Microconidia and macroconidia are numerous, and spiral hyphae are occasionally present. The primary differential diagnosis is *T. mentagrophytes*. In our case, the culture on SDA showed white colonies with peripherally radiating, centrally raised, and powdery margins. The slides culture revealed filamentous and spiral hyphae with a grape-like arrangement of microconidia laterally and terminally inserting at the hyphae. Using morphology identification of the isolate, we hardly distinguished it from *T. mentagrophytes*. Molecular identification is the best way of identification. Though the in house instrument such as PCR instrument is a high capital cost, it is still an inexpensive assay with high specificity. In our case, the diagnosis of *T. benhamiae* infection was made through molecular methods. The incidence rate of *T. benhamiae* might be severely underestimated in China, considering the high possibility of a missed diagnosis due to morphology identification, the unusual use of molecular identification in the clinic, and no previous report of *T. benhamiae*.

The treatment of *T. benhamiae* infection was consistent with other dermatophytoses [1]. Terbinafine is the first line of choice, with fluconazole and itraconazole being valid alternatives. In our case, the isolate was susceptible to all the tested antifungal drugs, and oral terbinafine treatment was sufficient (Fig. 1b).

In conclusion, *T. benhamiae* is an emerging zoophilic dermatophyte with an underestimated infection rate. It can cause highly inflammatory human infections, especially in children in contact with the fur of small animals. To avoid misdiagnosis with *M. canis* or *T. mentagrophytes*, specific ITS-based PCR for *T. benhamiae* identification might be necessary. Once diagnosed, the use of terbinafine is highly recommended to achieve optimal outcomes.

## Data Availability

All data generated or analyzed during this study are included in this published article. The sequence data have been deposited in the GenBank database (http://www.ncbi.nlm.nih.gov/Genbank/index.html) with the accession number MN536486.

## Abbreviations

CAS: Caspofungin
FLZ: Fluconazole
ITC: Itraconazole
MIC: Minimum inhibitory concentration
POS: Posaconazole
SDA: Sabouraud’s dextrose agar
TRB: Terbinafine
VRC: Voriconazole
